# Renal and cardiovascular outcomes and safety of SGLT2 inhibitors in patients aged ≥ 65 years with non-dialysis chronic kidney disease: a systematic review and meta-analysis of subgroup-level estimates derived from randomized trials

**DOI:** 10.1186/s12872-026-06248-z

**Published:** 2026-07-09

**Authors:** Ziyang Bao, Jingjia Hu, Yue Sun, Xianfa Li, Riyang Lin, Caifeng Zhu, Jiazhen Yin

**Affiliations:** Department of Nephrology, Hangzhou TCM Hospital Affiliated to Zhejiang Chinese Medical University, Hangzhou, 310007 China

**Keywords:** Chronic kidney disease, SGLT2 inhibitors, Elderly, Meta-analysis

## Abstract

**Background:**

Elderly patients with non-dialysis chronic kidney disease have high cardiorenal risk. SGLT2 inhibitors are considered to potentially provide combined renal and cardiac benefits.

**Objective:**

To evaluate renal and cardiovascular outcomes and safety of SGLT2 inhibitors in patients aged ≥ 65 years with non-dialysis chronic kidney disease.

**Methods:**

Randomized controlled trials from database inception to 2025-7-18 were systematically searched, and trials enrolling non-dialysis chronic kidney disease and reporting a ≥ 65-year subgroup were included. The primary outcome was an adverse renal composite endpoint, and the key secondary outcome was a composite of cardiovascular death or heart failure hospitalization. Safety focused on genital mycotic infections and volume depletion/hypotension, and acute kidney injury (AKI) and diabetic ketoacidosis (DKA) were summarized descriptively. Random-effects models based on log HR or RR (REML, Hartung–Knapp) were used, pre-specified sensitivity analyses were performed, and the certainty of evidence was assessed using GRADE.

**Results:**

Three double-blind trials including 5,831 patients aged ≥ 65 years were included. SGLT2 inhibitors reduced the risk of the adverse renal composite endpoint (HR = 0.68, 95% CI 0.52–0.89; I²=29.98%), and the risk of the composite of cardiovascular death or heart failure hospitalization (HR = 0.74, 95% CI 0.64–0.86; I²=35.53%). For safety, the risk of genital mycotic infections was increased (RR = 2.40, 95% CI 1.96–2.94; I²=23.85%), and the risk of volume depletion/hypotension was also increased (RR = 1.28, 95% CI 1.09–1.50; I²=0.00%); no signal of increased AKI was observed; DKA was very rare. GRADE rated the certainty of evidence as high for the renal outcome and moderate for the heart failure-related outcome.

**Conclusion:**

In patients aged ≥ 65 years with non-dialysis chronic kidney disease, SGLT2 inhibitors added to standard therapy achieve dual renal and cardiovascular benefits with a manageable safety profile, supporting their structured use with stratified monitoring. It is recommended that SGLT2 inhibitors be routinely included in treatment.

## Introduction

In elderly populations, the burden of chronic kidney disease is substantial, and the disease course is intertwined with metabolic comorbidities, blood pressure abnormalities, and difficulties in volume management, with renal outcomes and cardiovascular events mutually driving each other [1, 2]. In patients aged ≥ 65 years in the non-dialysis stage, it is necessary both to delay the progression of kidney function and to reduce the burden related to heart failure [3]. Sodium-glucose cotransporter 2 (SGLT2) inhibitors act through proximal tubular mechanisms to influence glomerular hemodynamics and improve volume and metabolic status, and have shown dual renal and cardiovascular protective signals in multiple populations [4, 5]. The clinical focus most relevant to elderly patients with non-dialysis chronic kidney disease is whether reproducible, age-compatible net clinical benefit can be achieved on top of standard kidney-protective therapy. Evidence centered on time-to-event endpoints is relatively sufficient in the overall population, whereas systematic evaluation targeting the ≥ 65-year subgroup remains insufficient [6]. Most existing studies are based mainly on the overall sample, with elderly subgroup analyses often appearing in supplementary materials, and the reporting pathways and endpoint definitions are inconsistent, making it difficult to directly compare effects and safety across trials [7]. The confirmation thresholds and components of renal composite endpoints differ among trials, the definitions of heart failure outcomes vary between composite endpoints and hospitalization, and elderly-specific volume-related adverse events and genital infections lack structured presentation [8]. In clinical decision-making, a quantitative evidence framework specifically for patients aged ≥ 65 years with non-dialysis chronic kidney disease is still lacking to determine whether SGLT2 inhibitors should be used as foundational therapy [9] and to plan monitoring and combination strategies accordingly. A rigorous synthesis of elderly subgroups in randomized controlled trials is therefore needed, with harmonized outcome definitions and effect size scales, to provide a practical profile of dual renal and cardiovascular benefits and risks. This study focuses on the ≥ 65-year subgroup in dedicated randomized controlled trials of chronic kidney disease, prespecifies clear research questions and outcome definitions, uses random-effects meta-analysis of log hazard ratios with Hartung–Knapp correction, and combines the Grading of Recommendations Assessment, Development and Evaluation (GRADE) to assess the certainty of evidence, in order to quantify the effects of SGLT2 inhibitors on the adverse renal composite endpoint and heart failure-related endpoints, and to delineate a safety profile centered on genital mycotic infections and volume depletion or hypotension. The results suggest that dual renal and cardiovascular benefits coexist with manageable risks in elderly patients with non-dialysis chronic kidney disease, providing a basis for incorporating this drug on top of standard therapy and implementing stratified monitoring.

## Materials and methods

### Study design and reporting standards

This study was a systematic review and meta-analysis subgroup-level estimates derived from randomized trials, with the study protocol prespecified and the analysis plan fixed before data extraction began. Subgroup analyses in meta-analyses are inherently limited and should be interpreted with caution. First, subgroup comparisons are observational and not based on randomized grouping, which precludes definitive causal inference. Second, conducting multiple subgroup comparisons increases the risk of type I error inflation due to repeated statistical testing. Third, subgroup meta-analyses generally yield lower statistical credibility and precision, as residual heterogeneity and reduced sample size can destabilize effect estimates. The reporting of the study followed the PRISMA 2020 Statement. The study population was restricted to patients aged ≥ 65 years with non-dialysis chronic kidney disease, the intervention was SGLT2 inhibitors, and the control was placebo or standard therapy.

### Literature search strategy

A systematic search was conducted in MEDLINE (via PubMed), Embase, Cochrane Central Register of Controlled Trials, Web of Science Core Collection, and ClinicalTrials.gov. The search period was from database inception to July 18, 2025. No language restrictions were applied. The reference lists of included studies and relevant reviews were searched retrospectively to identify additional records. The search strategy was constructed based on three conceptual groups of subject headings and free-text terms: drug (SGLT2 inhibitors), disease (chronic kidney disease), and study type (randomized controlled trial), with an additional restriction to elderly populations. Using PubMed as an example, the core search string was: ((“Sodium-Glucose Transporter 2 Inhibitors“[Mesh] OR “sodium-glucose cotransporter 2” OR SGLT2 OR dapagliflozin OR empagliflozin OR canagliflozin OR ertugliflozin) AND (“Kidney Diseases, Chronic“[Mesh] OR “chronic kidney disease” OR CKD OR “renal insufficiency, chronic” OR albuminuria OR proteinuria) AND (randomized OR randomised OR “Randomized Controlled Trial“[Publication Type]) AND (Aged[Mesh] OR elderly OR older OR “65 years”)). Search strings for each database were adapted in terms of fields and syntax within the same conceptual framework, and were calibrated for recall and accuracy through pilot searches and inspection of the results.

### Study inclusion and exclusion criteria

Inclusion criteria: ① randomized controlled trials; ② adult patients with chronic kidney disease who had not received dialysis at enrollment, with chronic kidney disease defined according to the criteria of the trial protocol; ③ the intervention was a single SGLT2 inhibitor plus background standard therapy, and the control was placebo or background standard therapy; ④ reported risk ratios for efficacy outcomes in the ≥ 65-year population or provided sufficient data to calculate an effect size; ⑤ a minimum follow-up duration of no less than 6 months; ⑥ reported at least one prespecified primary or key secondary outcome. Exclusion criteria: ① non-randomized studies, crossover trials, or single-arm studies; ② populations on maintenance dialysis or with kidney transplantation at baseline; ③ trials using dual SGLT1/2 inhibitors; ④ trials that did not report results for the ≥ 65-year subgroup and from which an effect size could not be derived; ⑤ trials that lacked prespecified outcomes or reported only incompatible measures; ⑥ duplicate publications, for which the report with the most complete data and the longest follow-up was used.

### Study outcomes and definitions

#### Primary outcome

The primary analysis used the adverse renal composite endpoint as prespecified in each trial. This composite endpoint consisted of one or more of the following components: sustained decline in estimated glomerular filtration rate reaching or exceeding a prespecified magnitude, doubling of serum creatinine, initiation of renal replacement therapy (hemodialysis, peritoneal dialysis, or kidney transplantation), and kidney-related death. The confirmation interval for the component of “sustained” was defined according to each trial protocol. Sensitivity analysis was restricted to definitions that included renal replacement therapy or kidney-related death.

#### Key secondary outcome

Priority was given to the composite of cardiovascular death or heart failure hospitalization. If a trial reported only heart failure hospitalization, it was included in the pooled analysis of heart failure hospitalization, and the impact of different definitions was examined in sensitivity analyses.

#### Safety outcomes

Safety outcomes were expressed as risk ratios calculated from the number of participants with adverse events and the total number of participants. Genital mycotic infections were identified according to investigator judgment or the corresponding terms in the adverse event coding system. Volume depletion or hypotension was determined by investigator judgment and aggregated using prespecified adverse event terms. Acute kidney injury preferably followed the Kidney Disease: Improving Global Outcomes (KDIGO) creatinine criteria; if a trial used different diagnostic criteria, the definition in that trial report was used. Diabetic ketoacidosis was defined based on confirmation of events by the trial team.

### Study screening and data extraction

Two researchers independently performed an initial screening of titles and abstracts, followed by full-text rescreening, using reference management software for de-duplication with manual verification. Reasons for exclusion were recorded item by item in the PRISMA flow diagram. Disagreements were resolved by a third researcher. Two researchers independently extracted data using a standardized prespecified form and cross-checked the results. The extracted items included basic trial characteristics, baseline data of participants, intervention and control regimens, sample size and number of events in the ≥ 65-year subgroup, risk ratios and 95% confidence intervals for each prespecified outcome, follow-up duration, descriptions of analysis sets, and risk of bias information. For time-to-event outcomes, hazard ratios and their 95% confidence intervals were uniformly extracted and converted to log hazard ratios and standard errors (standard errors were calculated as (ln upper limit − ln lower limit)/3.92) [10]. If a study did not provide a hazard ratio for the ≥ 65-year subgroup and an effect size could not be derived by appropriate methods, that study was not included in the quantitative synthesis for that outcome. For multi-arm trials with multiple SGLT2 inhibitor doses, the relevant intervention arms were first combined into a single intervention arm within the study to avoid double-counting.

### Quality assessment and risk of bias judgment

The Cochrane RoB 2 tool was used at the outcome level to assess five domains: randomization process, deviations from intended interventions, missing outcome data, outcome measurement, and selective reporting, with judgments of low risk, some concerns, or high risk [11]. Two researchers independently performed the assessments and reached agreement, with a third researcher adjudicating when necessary. The results of the risk of bias assessment were used in sensitivity analyses to test robustness.

### Statistical analysis

Statistical analyses were performed using R software (version 4.3), with the main packages including metafor and meta, and graphical outputs included the PRISMA flow diagram, forest plots, and risk of bias summary plots. Publication bias analyses and funnel plots were not presented because the number of studies was less than 10. The certainty of evidence for the primary outcome and heart failure-related outcomes was assessed using the GRADE approach [12], grading across five domains (limitations of studies, inconsistency of results, indirectness, imprecision, and publication bias), and a summary table was prepared to present the evidence level and reasons for downgrading.

### Effect size selection and pooling methods

For the primary outcome and heart failure-related outcomes, hazard ratios were used as the effect measure and were pooled using inverse-variance–weighted random-effects models based on log hazard ratios, with variance estimated by restricted maximum likelihood and confidence intervals adjusted using the Hartung–Knapp method. For safety outcomes, risk ratios were used as the effect measure and were pooled using random-effects models. When a study had a single zero event in one arm for a given outcome, a continuity correction of 0.5 was applied; studies with zero events in both arms for an outcome were excluded from the pooled analysis for that outcome [13]. All tests were two-sided, with a significance threshold set at 0.05, and 95% confidence intervals and 95% prediction intervals were reported. Because the number of studies was 3, subgroup analyses and meta-regression were not performed.

### Heterogeneity assessment and handling

Heterogeneity was quantified using Cochrane’s Q statistic, τ², and I², and was presented in forest plots. Exploration of the sources of heterogeneity was preferentially conducted through prespecified sensitivity analyses. I² and τ² were interpreted as descriptive indicators and were not used as threshold values to replace clinical judgment.

### Sensitivity analyses and narrative synthesis strategies

Two sensitivity analyses were prespecified in the protocol: ① a narrow-definition analysis of the adverse renal composite endpoint, including only studies whose composite endpoint contained renal replacement therapy or kidney-related death; ② repeating the pooling after excluding studies judged to be at high risk of bias for that outcome. For heart failure-related outcomes, when there were inconsistencies across studies between the definitions of the “composite of cardiovascular death or heart failure hospitalization” and “heart failure hospitalization”, the primary analysis prioritized the composite endpoint, and in sensitivity analyses, studies that reported only heart failure hospitalization were excluded. For outcomes that did not meet the quantitative pooling condition of “at least two studies with consistent effect measures”, a structured narrative synthesis was conducted, reporting the direction of effect, point estimates, and ranges of uncertainty, and explaining the reasons for not performing a meta-analysis.

## Results

### Literature search and study inclusion

**Fig. 1:**
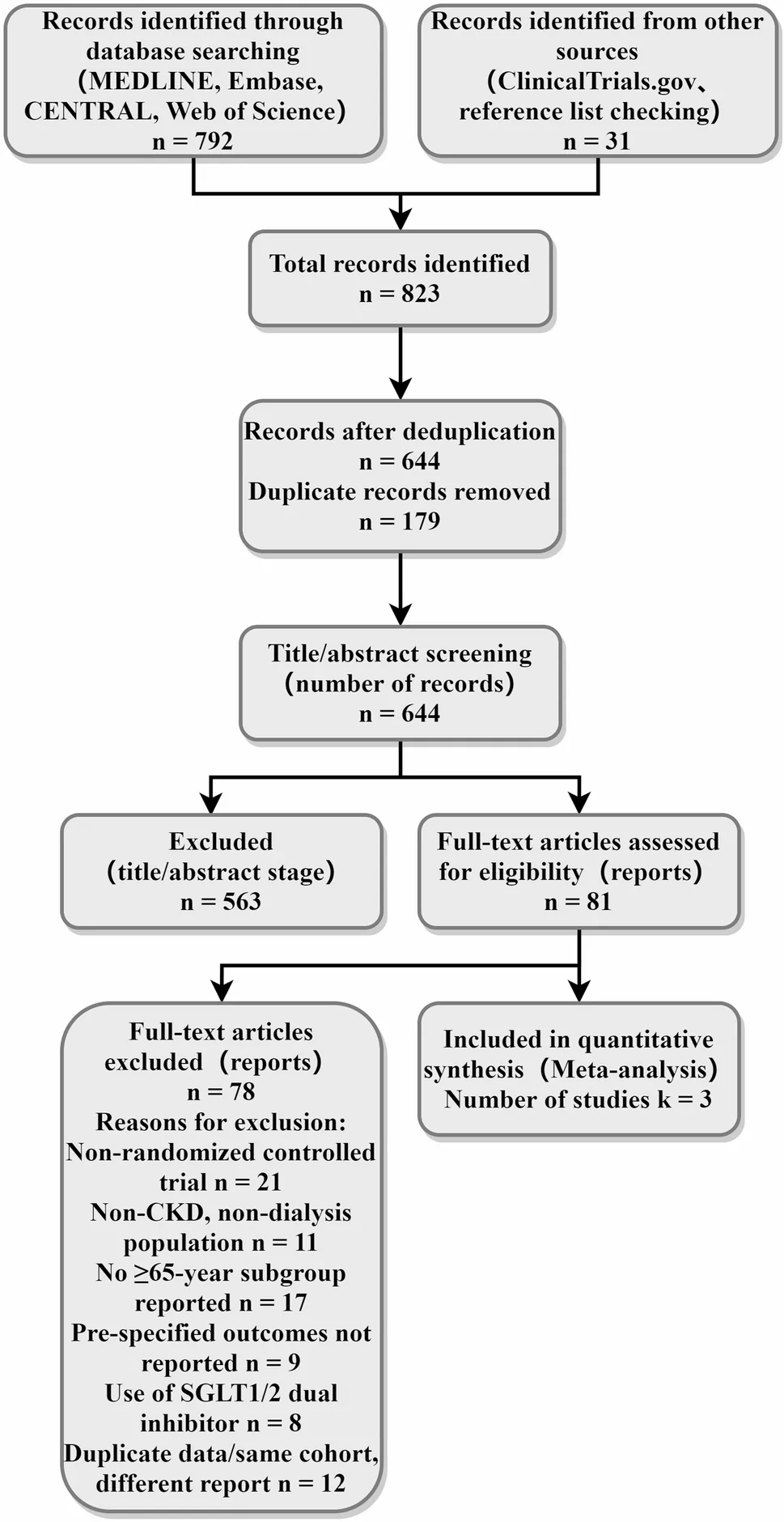
PRISMA literature screening flow diagram

A total of 823 records were identified. After de-duplication using software and manual checking, 644 records entered title and abstract screening. In the initial screening, 563 records were excluded, and 81 articles entered full-text assessment, of which 78 were excluded because of inappropriate study design, populations not consisting of non-dialysis CKD, no reporting of ≥ 65-year subgroup or prespecified outcomes, use of dual SGLT1/2 inhibitors, or duplicate publication. Finally, only 3 randomized controlled trials (Heerspink HJL 2020, PMID: 32970396; Herrington WG 2023, PMID: 36331190; Perkovic V 2019, PMID: 30990260) were included in the meta-analysis (Fig. 1).

### Characteristics of included studies and populations

Three randomized, double-blind, controlled trials were included, with a total of 5,831 patients aged ≥ 65 years. The mean age was approximately 71 years, and about one-third were women. Baseline eGFR was mostly in the range of 36–46 mL/min/1.73 m², UACR was generally elevated, and the proportions of type 2 diabetes and use of renin–angiotensin system inhibitors were both high, indicating a substantial overall burden of renal and cardiovascular risk in the population (Table 1).

**Table 1:** Summary of trial design and baseline characteristics of included randomized controlled trials and ≥ 65-year subgroups

Characteristic	Heerspink HJL 2020;DAPA-CKD [6]	Herrington WG 2023;EMPA-KIDNEY [8]	Perkovic V 2019;CREDENCE [25]
Study design	Randomized, double-blind, placebo-controlled, parallel-group	Randomized, double-blind, placebo-controlled, parallel-group	Randomized, double-blind, placebo-controlled, parallel-group
Intervention and control	Dapagliflozin 10 mg once daily; placebo	Empagliflozin 10 mg once daily; placebo	Canagliflozin 100 mg once daily; placebo
Median follow-up (months)	28.74	23.86	30.31
CKD inclusion criteria	eGFR 25–75; UACR 200–5000	eGFR 20–<45; or eGFR 45–<90 and UACR ≥ 200	eGFR 30–<90 and UACR ≥ 300
Sample size of ≥ 65-year subgroup (n)	1637	2481	1713
Age (years)	71.42 ± 4.87	72.08 ± 5.12	71.06 ± 4.72
Female proportion (%)	36.28	37.91	34.57
Baseline eGFR (mL/min/1.73 m²)	41.78 ± 12.64	36.12 ± 11.27	45.88 ± 13.94
Baseline UACR (mg/g, M[IQR])	934 [412–1938]	648 [294–1473]	912 [427–1884]
Proportion of type 2 diabetes (%)	64.28	47.31	100
Use of renin–angiotensin system inhibitors (%)	96.83	86.72	98.21
Original kidney-related composite endpoint	Primary endpoint: sustained decline in eGFR ≥ 50%, ESKD, or renal/cardiovascular death; kidney-specific endpoint: sustained decline in eGFR ≥ 50%, ESKD, or renal death	Primary endpoint: kidney disease progression or cardiovascular death; kidney disease progression included ESKD, sustained eGFR < 10 mL/min/1.73 m², sustained decline in eGFR ≥ 40%, or renal death	Primary endpoint: ESKD, doubling of serum creatinine (Scr), or renal/cardiovascular death; kidney-specific endpoint: ESKD, doubling of Scr, or renal death
Kidney function decline component	Sustained decline in eGFR ≥ 50%	Sustained decline in eGFR ≥ 40%, or sustained decline to < 10 mL/min/1.73 m²	Doubling of Scr
Sustained/confirmation criteria	Confirmation by repeat measurement, interval ≥ 28 days	Two consecutive scheduled follow-up measurements, interval ≥ 30 days	Confirmation by repeat measurement, interval ≥ 30 days
Kidney failure / kidney replacement therapy component	Maintenance dialysis ≥ 28 days, kidney transplantation, or sustained eGFR < 15 mL/min/1.73 m²	Maintenance dialysis or kidney transplantation	Dialysis ≥ 30 days, kidney transplantation, or sustained eGFR < 15 mL/min/1.73 m²
Death component	The primary endpoint included renal death and cardiovascular death; the kidney-specific endpoint included renal death only	Kidney disease progression included renal death; the primary endpoint additionally included cardiovascular death	The primary endpoint included renal death and cardiovascular death; the kidney-specific endpoint included renal death only

### Risk of bias assessment

**Fig. 2:**
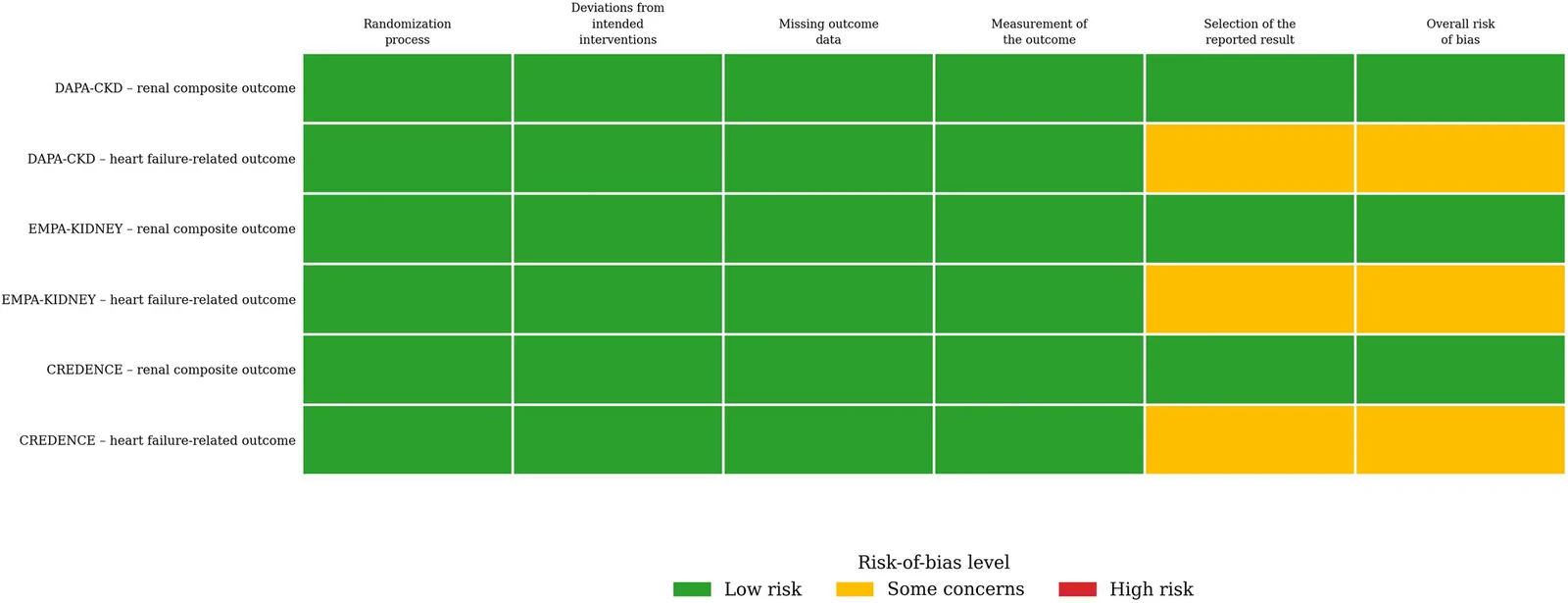
Risk of bias (RoB 2) traffic-light plot (by outcome)

According to the RoB 2 algorithm, the overall risk of bias for the renal composite outcomes in the three studies was judged to be low risk. For heart failure outcomes, the presence of some concerns in the domain of “selective reporting” led to an overall judgment of some concerns, while all other domains besides “selective reporting” were judged to be low risk (Fig. 2).

### Primary outcome

**Fig. 3:**
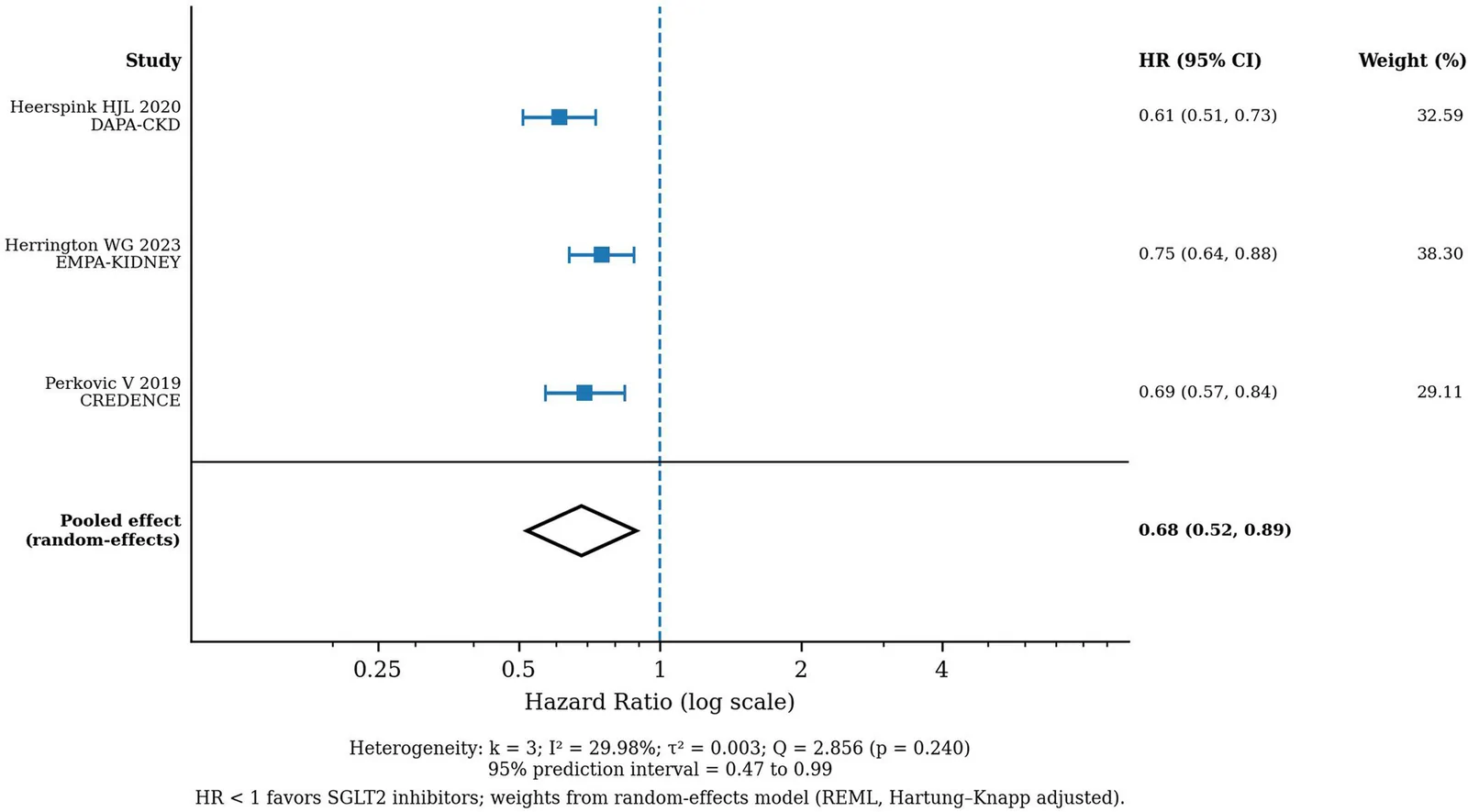
Forest plot of the meta-analysis for the primary outcome (adverse renal composite endpoint)

Using inverse-variance–weighted random-effects models based on log HRs, the analysis showed that in patients aged ≥ 65 years with non-dialysis chronic kidney disease, SGLT2 inhibitors significantly reduced the risk of the adverse renal composite endpoint, with a pooled HR of 0.68 (95% CI 0.52–0.89) and low heterogeneity (I²=29.98%) (Fig. 3). Using REML random-effects models with Hartung–Knapp correction, the pooled HR under the narrow definition was 0.66 (95% CI 0.49–0.89; I²=22.71%), and after excluding studies at high risk of bias, the pooled HR was 0.68 (95% CI 0.52–0.89; I²=29.98%), indicating robust results (Table 2).

**Table 2:** Summary of sensitivity analysis results for the primary outcome

Analysis scenario	Number of included studies (k)	Pooled HR	95% CI	95% PI	I²(%)	τ²	*p*
Narrow definition (including only renal replacement therapy or kidney-related death)	3	0.66	0.49–0.89	0.44–0.97	22.71	0.002	0.298
Excluding studies with overall high risk of bias	3	0.68	0.52–0.89	0.47–0.99	29.98	0.003	0.240

### Key secondary outcome

**Fig. 4:**
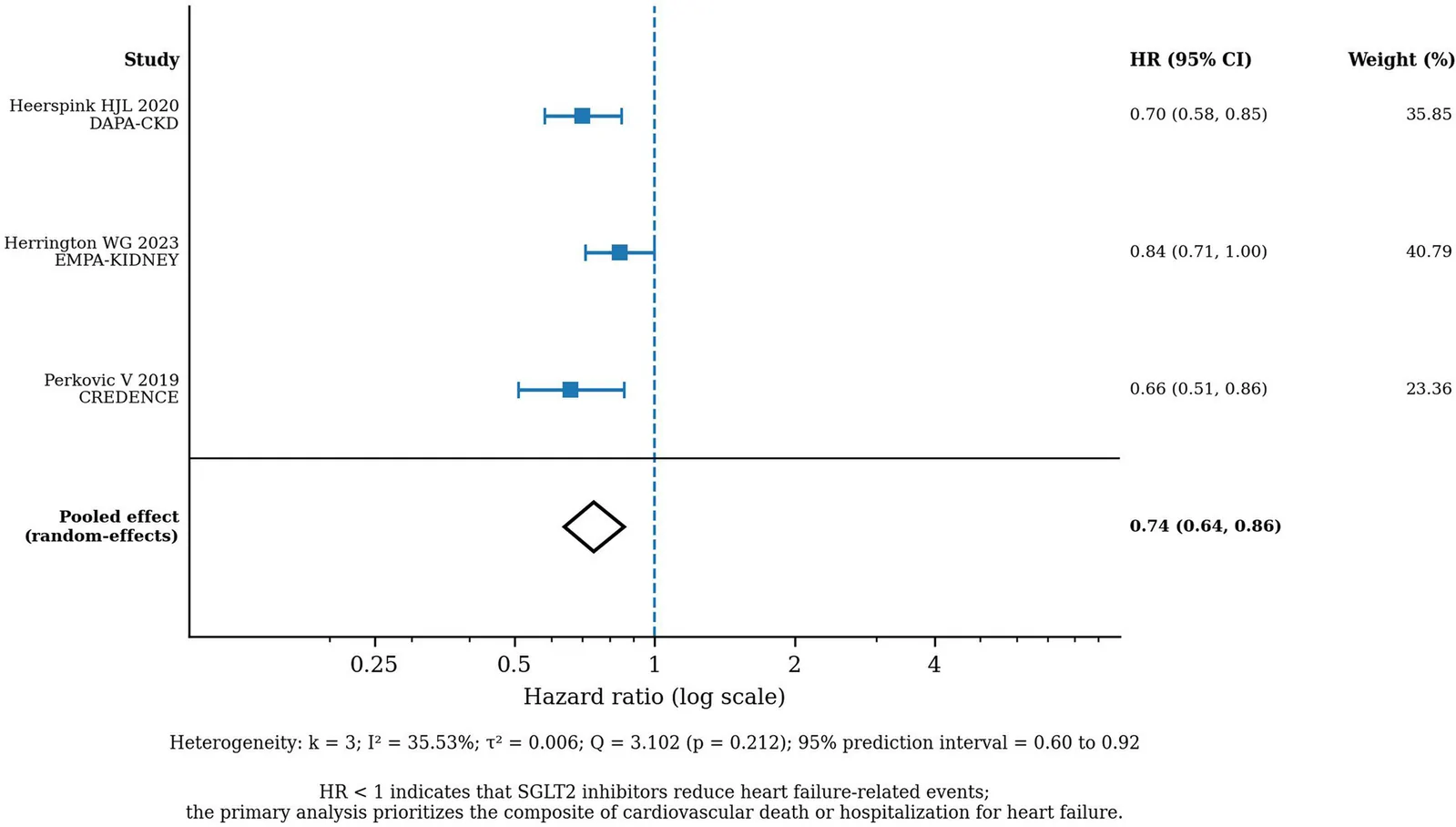
Meta-analysis forest plot of heart failure-related outcomes

Using inverse-variance–weighted random-effects models based on log HRs, the analysis showed that in patients aged ≥ 65 years with non-dialysis chronic kidney disease, SGLT2 inhibitors reduced the risk of the composite of cardiovascular death or heart failure hospitalization, with a pooled HR of 0.74 (95% CI 0.64–0.86) and moderate heterogeneity (I²=35.53%) (Fig. 4). Using REML random-effects models with Hartung–Knapp correction and excluding studies that reported only heart failure hospitalization, the pooled HR for the heart failure-related composite endpoint was 0.77 (95% CI 0.65–0.91), with lower heterogeneity (I²=27.46%; 95% PI 0.56–1.04) (Table 3).

**Table 3:** Summary of sensitivity analysis results for consistency of definitions of heart failure-related endpoints

Analysis scenario	Number of included studies (k)	Pooled HR	95% CI	95% PI	I²(%)	τ²	*p*
Excluding studies that reported only heart failure hospitalization and did not report the composite endpoint	2	0.77	0.65–0.91	0.56–1.04	27.46	0.004	0.196

This sensitivity analysis was used to examine the impact of different definitions of heart failure endpoints on the pooled results

### Safety outcomes

**Fig. 5:**
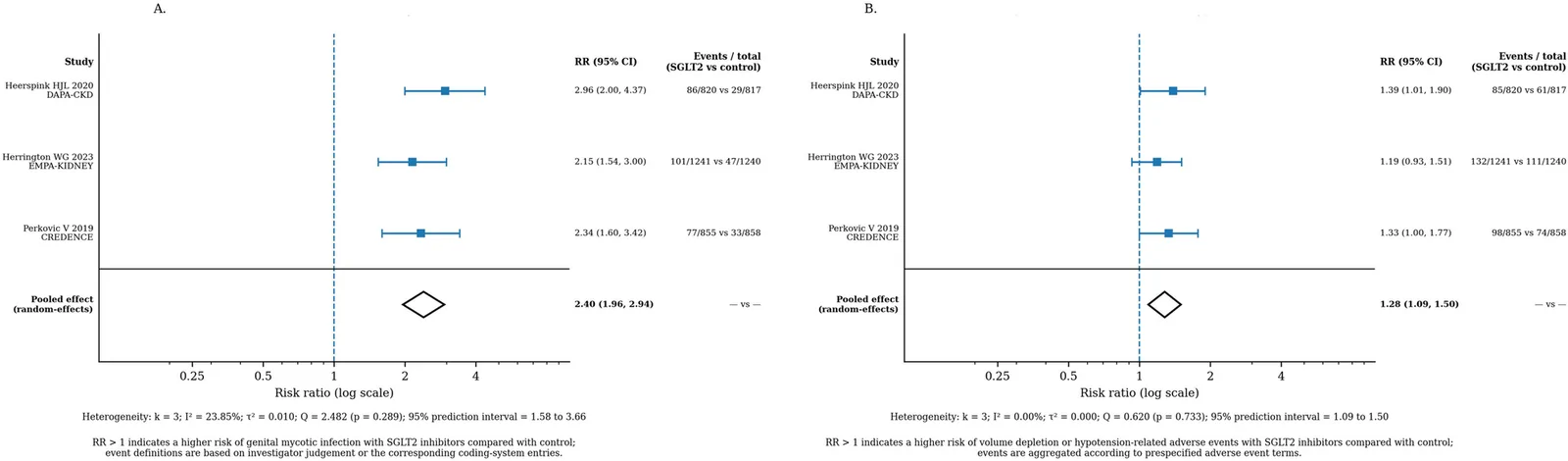
Safety outcomes of SGLT2 inhibitors. **A**: Meta-analysis forest plot of genital mycotic infections. **B**: Meta-analysis forest plot of volume depletion or hypotension-related adverse events

In patients aged ≥ 65 years with non-dialysis chronic kidney disease, inverse-variance–weighted random-effects models based on log RRs showed that the risk of genital mycotic infections was significantly increased in the SGLT2 inhibitor group, with a pooled RR of 2.40 (95% CI 1.96–2.94, I²=23.85%) (Fig. 5A). The risk of volume depletion or hypotension-related adverse events was also increased, with a pooled RR of 1.28 (95% CI 1.09–1.50, I²=0.00%) (Fig. 5B).

For the outcome of AKI, only DAPA-CKD and EMPA-KIDNEY reported data for the ≥ 65-year subgroup based on KDIGO creatinine criteria. In DAPA-CKD, AKI occurred in 38/820 patients in the SGLT2 group and 53/817 patients in the control group, with an RR of 0.71 (95% CI 0.48–1.07). In EMPA-KIDNEY, AKI occurred in 61/1241 patients in the SGLT2 group and 74/1240 patients in the control group, with an RR of 0.82 (95% CI 0.59–1.15). In both trials, the direction of effect was consistent and no increased risk of AKI was observed, suggesting a possible protective effect, although the confidence intervals all crossed 1. CREDENCE did not separately report AKI events for the ≥ 65-year subgroup, so a study-level RR could not be estimated and it was not included in quantitative pooling. For DKA, all three trials had no analyzable events or events were extremely few in the ≥ 65-year subgroup, so DKA can only be considered extremely rare among elderly patients with non-dialysis CKD; this study could not quantitatively assess its relative risk, and no clear safety signal was observed.

#### Assessment of certainty of evidence

Assessment using the GRADE approach showed that the level of evidence for the adverse renal composite endpoint was high, with no downgrading in any domain across the three studies including a total of 5,831 patients aged ≥ 65 years. The level of evidence for the heart failure-related composite endpoint was moderate, mainly because the risk of bias domain was downgraded by one level due to most subgroup results being reported in supplementary materials (Table 4).

**Table 4:** Summary of certainty of evidence for the primary outcome and heart failure-related endpoints

Item of assessment	Adverse renal composite endpoint	Heart failure-related endpoints
Number of studies (k)	3	3
Total number of participants (≥ 65-year subgroup)	5831	5831
Judgment in the risk of bias domain	No downgrading: 3/3 studies were at low risk of bias at the outcome level; randomized, double-blind, central adjudication of events, low loss to follow-up	Downgraded by one level: ≥65-year subgroup results were mostly presented in supplementary materials, with some concerns about selective reporting
Judgment on inconsistency	No downgrading: I²=29.98%, direction of effect consistent	No downgrading: I²=35.53%, direction consistent
Judgment on indirectness	No downgrading: population, intervention, control, and outcomes were consistent with the research question	No downgrading: endpoint definitions were similar and applicable to CKD patients aged ≥ 65 years
Judgment on imprecision	No downgrading: 95% CI did not cross the line of no effect; number of events was sufficient; 95% PI was close to the line of no effect but not enough to warrant downgrading	No downgrading: 95% CI did not cross the line of no effect
Handling of the publication bias domain	No downgrading: the small number of studies did not allow reliable assessment; no signals of discrepancies between registration and reporting were observed	No downgrading: the small number of studies, and no evidence suggesting publication bias favoring positive results was observed
Overall level of evidence	High	Moderate
Brief description of reasons for downgrading	No downgrading	Downgraded by one level due to risk of bias (selective reporting)

## Discussion

In patients aged ≥ 65 years with non-dialysis chronic kidney disease, SGLT2 inhibitors were associated with a reduced risk of the adverse renal composite endpoint, and the pooled effect indicated a clear reduction in relative risk with low heterogeneity. The results remained consistent when using a narrow definition of the endpoint or excluding studies at high risk of bias, and the GRADE assessment rated the level of evidence as high, indicating that the conclusions are stable and generalizable. Physiologically, inhibition of glucose and sodium transport in the proximal tubule increases distal chloride delivery, reactivates tubuloglomerular feedback, increases afferent arteriolar tone, reduces intraglomerular pressure, and slows the long-term rate of decline in filtration rate, accompanied by reductions in proteinuria [14–18]. At the same time, moderate osmotic diuresis, mild reductions in volume and blood pressure, decreases in uric acid, and attenuation of inflammatory and fibrotic signaling occur, forming a kidney-protective effect centered on hemodynamics and accompanied by improvements in metabolic and inflammatory status [19]. The early decline in filtration rate reflects hemodynamic resetting rather than structural kidney injury, which is consistent with the safety finding that no increased risk of acute kidney injury was observed [20]. Neuen et al. indicated that the renoprotective effects of SGLT2 inhibitors on slowing chronic kidney disease progression were consistent across all eGFR subgroups, and the corresponding HRs as follows: HR = 0.61 for eGFR ≥ 60 mL/min/1.73 m², HR = 0.57 for eGFR of 45 to < 60 mL/min/1.73 m², HR = 0.64 for eGFR of 30 to < 45 mL/min/1.73 m², and HR = 0.71 for eGFR < 30 mL/min/1.73 m², with a P value for trend of 0.16 [21]. Large-scale data encompassing more than 27 million participants from the CKD Prognosis Consortium further revealed that individuals with stage 4 CKD (eGFR < 30 mL/min/1.73 m²) carry a 110- to 580-fold higher risk of kidney failure compared with patients with preserved renal function (eGFR ≥ 90 mL/min/1.73 m²) and normal albuminuria, with risk increments stratified by albuminuria severity [22]. These findings strengthen the guideline recommendations from major cardiovascular and nephrology societies for initiating SGLT2 inhibitor treatment in patients with CKD and an eGFR of ≥ 20 mL/min/1.73 m², independent of diabetes mellitus status [23, 24]. Compared with younger individuals, older patients generally have diminished baseline renal reserve, slower age-related physiological eGFR decline, and a higher burden of comorbidities, which render renal outcomes highly sensitive to the threshold definition adopted. Such methodological heterogeneity across studies may distort between-study comparisons of SGLT2 inhibitor efficacy in elderly CKD cohorts. In recognition of this issue, we acknowledge that standardized renal endpoint definitions are essential to enhance the consistency and generalizability of future geriatric renal trials. Compared with the overall populations in previous CKD randomized controlled trials, the magnitude of the relative effect was similar, suggesting that age itself is not a major effect modifier [25]. In the high-risk elderly population included in this study, lower baseline filtration rates and higher levels of proteinuria correspond to higher event rates, and greater absolute numbers of events avoided can be expected. The prediction interval was close to but did not cross the line of no effect, indicating variability in effect size across different clinical settings but a consistent overall direction. This is in line with the renal protective trends previously observed across different levels of kidney function and proteinuria, and supports the use of SGLT2 inhibitors in combination with standard kidney-protective therapy to further reduce the risk of renal endpoints in elderly patients with non-dialysis chronic kidney disease.

In patients aged ≥ 65 years with non-dialysis chronic kidney disease, the incidence of the composite of cardiovascular death or heart failure hospitalization was observed to decrease, and the effect remained stable with reduced heterogeneity after harmonization of endpoint definitions, indicating the reproducibility of the results and methodological robustness. A recent study by Mariani et al. revealed that the use of SGLT2i induced a significant reduction of atrial and ventricular arrhythmias events in HFrEF carriers of implantable cardioverter defibrillator or cardiac resynchronization therapy defibrillator [26]. Indeed, several clinical trials, including landmark studies such as EMPA-REG OUTCOME, CANVAS Program, and DECLARE-TIMI 58, have demonstrated robust reductions in cardiovascular events, particularly heart failure hospitalizations and cardiovascular mortality, among patients treated with SGLT2i. However, subsequent trials showed that SGLT2i benefits extend beyond the diabetic population, encompassing individuals with and without diabetes. Additionally, SGLT2i exhibit nephroprotective effects, manifesting as a slowing of the progression of chronic kidney disease and a reduction in the risk of end-stage kidney disease. Another review showed that multiple clinical trials have clearly demonstrated substantial reductions in cardiovascular events, especially heart failure hospitalizations and cardiovascular mortality, among patients treated with SGLT2i. Moreover, SGLT2i show nephroprotective effects, which are manifested as a deceleration of the progression of chronic kidney disease and a decrease in the risk of end-stage kidney disease [27]. Beyond their well-established cardioprotective effects, SGLT2 inhibitors exert substantial improvements in endothelial function among individuals with diabetic cardiomyopathy, highlighting additional vasculoprotective benefits of this drug class [28]. The physiological basis can be explained by mild natriuresis and osmotic diuresis [29], with preferential mobilization of interstitial fluid, reductions in venous return and preload, improvement in pulmonary congestion and ventricular filling pressure, and alleviation of renal venous congestion [30], accompanied by modest reductions in blood pressure and body weight, decreased uric acid levels, and more efficient myocardial substrate utilization, thereby optimizing cardiorenal interaction from both volume and metabolic perspectives [31]. Elderly patients with chronic kidney disease have greater sodium sensitivity and higher venous pressure load, and small changes in volume management are more likely to elicit responses at the endpoint level, which is consistent with the observed decrease in the composite endpoint [32]. Compared with evidence from previous randomized trials in overall populations with heart failure or chronic kidney disease, the feature that benefits are mainly driven by heart failure-related events was reproduced, with the magnitude of effect falling within the previously reported range and no age-related reversal of effect direction observed [33]; after adopting stricter endpoint definitions, between-study variability was reduced, suggesting that differences are more likely attributable to endpoint definitions and the composition of events rather than inconsistency in drug effects [34]. Given the higher baseline risk and event density in elderly populations, the above relative effects are more likely to translate into greater absolute benefits, supporting the incorporation of SGLT2 inhibitors into treatment pathways aimed at reducing heart failure-related burden on the basis of optimized natriuresis, diuresis, and blood pressure control, and the use of individualized fluid management to reduce volume-related adverse events.

Regarding safety, the risk profile of SGLT2 inhibitors shows predictable and overall manageable characteristics. Genital mycotic infections and volume depletion- or hypotension-related adverse events occur more frequently and represent clear pharmacological extensions, and in clinical practice they mostly present as mild to moderate events that can be prevented and managed by reinforcing local hygiene, early recognition of pruritus or changes in secretions, assessment of changes in blood pressure and body weight, and individualized adjustment of diuretic and antihypertensive regimens. Considering the potential adverse effects of SGLT2 inhibitors, including genital infection and hypotension, targeted clinical management is essential. Clinicians should carry out systematic patient education to help patients recognize early symptoms of infection and hypotension. Meanwhile, the frequency of follow-up visits should be appropriately increased during treatment, so as to timely identify and intervene relevant adverse reactions. No increasing trend in the risk of acute kidney injury was observed, and the direction of effect even tended to be risk reduction, which is consistent with the understanding that the early modest decline in eGFR mainly reflects resetting of intraglomerular pressure rather than acute nephron injury [35], and is also in line with the direction of benefit on the adverse renal composite endpoint. Events of diabetic ketoacidosis in the existing ≥ 65-year subgroup were rarely recorded and are difficult to assess quantitatively, but at least no recognizable persistent safety signal has emerged, suggesting that in elderly populations mainly consisting of non-dialysis chronic kidney disease, this complication is more likely to be confined to special situations such as severe stress, insulin therapy, or severe dehydration [36]. Previous randomized trials in populations with different underlying diseases and age groups have consistently reported an increase in fungal infections and volume-related events, without confirming a systematic increase in AKI [37], and the findings of this study are consistent with this pattern. Against the background of clear benefits in both renal and heart failure outcomes, this risk profile, dominated by local infections and volume-related events, supports achieving dual renal and cardiovascular protection for elderly patients with non-dialysis chronic kidney disease in clinical practice through standardized patient education, close monitoring, and careful management of concomitant medications, without substantially increasing irreversible serious adverse events.

Several key limitations inherent to the present meta-analysis should be acknowledged. First, the analysis was restricted to only three eligible trials, and the reliance on aggregated study-level data precluded adjustment for individual-level heterogeneity, thereby introducing potential selective reporting bias. Second, incomplete reporting of several prespecified subgroup indicators across the included studies limited our ability to evaluate the impacts of advanced age (≥ 75 years), severely reduced eGFR, frailty status, and concomitant diuretic dosage and blood pressure targets on therapeutic efficacy and safety outcomes. Such data insufficiency may result in unstable confidence intervals, less reliable prediction intervals, and low precision of heterogeneity estimates, which compromise the robustness and comprehensiveness of our subgroup findings. Third, inconsistent reporting of composite heart failure endpoints and the predominant presentation of elderly subgroup data in supplementary materials further increase the risk of selective reporting uncertainty. In addition, the low incidence of diabetic ketoacidosis and certain safety events, together with the lack of age-stratified data for patients aged ≥ 65 years, prohibited rigorous quantitative subgroup evaluation. Finally, all subgroup comparisons were observational in nature without within-subgroup randomization. Collectively, these methodological limitations necessitate cautious interpretation of the current subgroup results, and future studies with more complete individual-level and stratified data are warranted to validate our findings.

Also, the median follow-up duration was approximately 24–30 months, which is not sufficient to evaluate very long-term hard endpoints and rare adverse events. To improve the usability of the evidence, it is recommended to conduct individual participant data meta-analyses centered on patients aged ≥ 65 years, with harmonized prespecified definitions of renal composite and heart failure composite endpoints, and stratification by age and frailty, eGFR and UACR, history of heart failure, and diuretic intensity, reporting absolute risks, NNT, and NNH; to establish prospective registries for elderly CKD, standardize safety terminology and event adjudication, and fully report elderly strata; to embed strategies for coordinated volume management and blood pressure lowering in real-world studies and pragmatic trials, in order to verify net clinical benefit and optimal monitoring frequency across different baseline risk strata; and to improve data sharing and preregistration mechanisms, prespecify narrow renal composite endpoints and endpoint-consistency sensitivity analyses, and collect quality control indicators such as methods of event adjudication and loss to follow-up, so as to enhance cross-study comparability and the external validity of conclusions.

## Conclusion

In patients aged ≥ 65 years with non-dialysis chronic kidney disease, SGLT2 inhibitors on top of standard therapy provide clear renal benefits and reduce heart failure-related events, and the effects remain robust across different endpoint definitions and methodological tests. Overall safety is acceptable, with the main risks being genital mycotic infections and volume depletion or hypotension, with no increase in acute kidney injury and rare diabetic ketoacidosis. Across trials with different proportions of participants with type 2 diabetes, the direction of benefit was generally consistent; however, effect modification by diabetes status could not be formally evaluated. It is recommended that SGLT2 inhibitors be routinely incorporated into treatment after assessment of volume status and concomitant medications, with close monitoring for genital infection and hypotension is required during medication, and optimization of diuretic and RAS inhibitor doses.

## Data Availability

The datasets used and/or analysed during the current study are available from the corresponding author (Jiazhen Yin) on reasonable request.

## Abbreviations

SGLT2: Sodium-glucose cotransporter 2
AKI: acute kidney injury
DKA: diabetic ketoacidosis
KDIGO: Kidney Disease: Improving Global Outcomes
eGFR: estimated glomerular filtration rate

## References

[1] Brady BM, Suffoletto JA, Sankary R, Chertow GM. Chronic kidney disease in older adults: challenges and opportunities for the primary care provider. BMC Prim Care. 2024;25(1):388. Published 2024 Nov 1. 10.1186/s12875-024-02638-4 PMC11529074 39487419

[2] Jarocki M, Green S, Wu HHL, Chinnadurai R. Cardiorenal Syndrome in the Elderly: Challenges and Considerations. Geriatr (Basel). 2025;10(4):104. 10.3390/geriatrics10040104. Published 2025 Aug 4. PMC12385558 40863571

[3] Zoccali C, Levin A, Mallamaci F, Giugliano R, De Caterina R. Advanced chronic kidney disease coexisting with heart failure: navigating patients’ management. Clin Kidney J. 2025;18(5):sfaf128. 10.1093/ckj/sfaf128. Published 2025 May 2. PMC12089769 40395996

[4] Koh ES, Kim GH, Chung S. Intrarenal Mechanisms of Sodium-Glucose Cotransporter-2 Inhibitors on Tubuloglomerular Feedback and Natriuresis. Endocrinol Metab (Seoul). 2023;38(4):359–72. 10.3803/EnM.2023.1764. PMC10475968 37482684

[5] Wang M, Zuo L. Cardiorenal Benefits of SGLT2 Inhibitors in Patients with Chronic Kidney Disease and Concomitant Hypertension. Cardiorenal Med. 2025;15(1):496–509. 10.1159/000545622. 40258345

[6] Heerspink HJL, Stefánsson BV, Correa-Rotter R, et al. Dapagliflozin in Patients with Chronic Kidney Disease. N Engl J Med. 2020;383(15):1436–46. 10.1056/NEJMoa2024816. 32970396

[7] Aldafas R, Crabtree T, Alkharaiji M, Vinogradova Y, Idris I. Sodium-glucose cotransporter-2 inhibitors (SGLT2) in frail or older people with type 2 diabetes and heart failure: a systematic review and meta-analysis. Age Ageing. 2024;53(1):afad254. 10.1093/ageing/afad254. PMC10825241 38287703

[8] The EMPA-KIDNEY, Collaborative Group, Herrington WG, Staplin N, et al. Empagliflozin in Patients with Chronic Kidney Disease. N Engl J Med. 2023;388(2):117–27. 10.1056/NEJMoa2204233. PMC7614055 36331190

[9] Liabeuf S, Minutolo R, Floege J, Zoccali C. The use of SGLT2 inhibitors and GLP-1 receptor agonists in older patients: a debate on approaches in CKD and non-CKD populations. Clin Kidney J. 2024;18(2):sfae380. 10.1093/ckj/sfae380. Published 2024 Dec 2. PMC11788569 39906070

[10] Tierney JF, Stewart LA, Ghersi D, Burdett S, Sydes MR. Practical methods for incorporating summary time-to-event data into meta-analysis. Trials. 2007;8:16. 10.1186/1745-6215-8-16. Published 2007 Jun 7. PMC1920534 17555582

[11] Sterne JAC, Savović J, Page MJ, et al. RoB 2: a revised tool for assessing risk of bias in randomised trials. BMJ. 2019;366:l4898. 10.1136/bmj.l4898. 31462531

[12] Guyatt GH, Oxman AD, Vist GE, et al. GRADE: an emerging consensus on rating quality of evidence and strength of recommendations. BMJ. 2008;336(7650):924–6. 10.1136/bmj.39489.470347.AD PMC2335261 18436948

[13] Bradburn MJ, Deeks JJ, Berlin JA, Russell Localio A. Much ado about nothing: a comparison of the performance of meta-analytical methods with rare events. Stat Med. 2007;26(1):53–77. 10.1002/sim.2528. 16596572

[14] Naaman SC, Bakris GL. Diabetic Nephropathy: Update on Pillars of Therapy Slowing Progression. Diabetes Care. 2023;46(9):1574–86. 10.2337/dci23-0030. PMC10547606 37625003

[15] Mantsiou C, Karagiannis T, Kakotrichi P, et al. Glucagon-like peptide-1 receptor agonists and sodium-glucose co-transporter-2 inhibitors as combination therapy for type 2 diabetes: A systematic review and meta-analysis. Diabetes Obes Metab. 2020;22(10):1857–68. 10.1111/dom.14108. 32476254

[16] Neuen BL, Heerspink HJL, Vart P, et al. Estimated Lifetime Cardiovascular, Kidney, and Mortality Benefits of Combination Treatment With SGLT2 Inhibitors, GLP-1 Receptor Agonists, and Nonsteroidal MRA Compared With Conventional Care in Patients With Type 2 Diabetes and Albuminuria. Circulation. 2024;149(6):450–62. 10.1161/circulationaha.123.067584. 37952217

[17] Sen T, Heerspink HJL. A kidney perspective on the mechanism of action of sodium glucose co-transporter 2 inhibitors. Cell Metab. 2021;33(4):732–9. 10.1016/j.cmet.2021.02.016. 33691091

[18] van Bommel EJM, Muskiet MHA, van Baar MJB, et al. The renal hemodynamic effects of the SGLT2 inhibitor dapagliflozin are caused by post-glomerular vasodilatation rather than pre-glomerular vasoconstriction in metformin-treated patients with type 2 diabetes in the randomized, double-blind RED trial. Kidney Int. 2020;97(1):202–12. 10.1016/j.kint.2019.09.013. 31791665

[19] Hou YC, Zheng CM, Yen TH, Lu KC. Molecular Mechanisms of SGLT2 Inhibitor on Cardiorenal Protection. Int J Mol Sci. 2020;21(21):7833. Published 2020 Oct 22. 10.3390/ijms21217833 PMC7660105 33105763

[20] Zhuo M, Paik JM, Wexler DJ, Bonventre JV, Kim SC, Patorno E. SGLT2 inhibitors and the risk of acute kidney injury in older adults with type 2 diabetes. Am J Kidney Dis. 2022;79(6):858–e8671. 10.1053/j.ajkd.2021.09.015. PMC9079190 34762974

[21] Neuen BL, Fletcher RA, Anker SD, et al. SGLT2 Inhibitors and Kidney Outcomes by Glomerular Filtration Rate and Albuminuria: A Meta-Analysis. JAMA. 2026;335(3):233–44. 10.1001/jama.2025.20834. PMC12595549 41203232

[22] Grams ME, Coresh J, Matsushita K, et al. Estimated Glomerular Filtration Rate, Albuminuria, and Adverse Outcomes: An Individual-Participant Data Meta-Analysis. JAMA. 2023;330(13):1266–77. 10.1001/jama.2023.17002. PMC10548311 37787795

[23] McDonagh TA, Metra M, Adamo M, et al. 2023 Focused Update of the 2021 ESC Guidelines for the diagnosis and treatment of acute and chronic heart failure. Eur Heart J. 2023;44(37):3627–39. 10.1093/eurheartj/ehad195. 37622666

[24] Chronic Kidney Disease and Risk Management. Standards of Care in Diabetes-2025. Diabetes Care. 2025;48(1 Suppl 1):S239–51. 10.2337/dc25-S011. PMC11635029 39651975

[25] Perkovic V, Jardine MJ, Neal B, et al. Canagliflozin and Renal Outcomes in Type 2 Diabetes and Nephropathy. N Engl J Med. 2019;380(24):2295–306. 10.1056/NEJMoa1811744. 30990260

[26] Mariani MV, Lavalle C, Palombi M, et al. SGLT2i reduce arrhythmic events in heart failure patients with cardiac implantable electronic devices. ESC heart Fail. 2025;12(3):2125–33. 10.1002/ehf2.15223. PMC12055389 39921334

[27] Cersosimo A, Drera A, Adamo M, et al. Exploring the Cardiorenal Benefits of SGLT2i: A Comprehensive Review. Kidney and Dialysis. 2024;4(4):184-202. 10.3390/kidneydial4040016.

[28] Cersosimo A, Amore L, Cimino G, et al. Impact of SGLT2 inhibitors on endothelial function and echocardiographic parameters in dilated cardiomyopathy. J Cardiovasc Med (Hagerstown Md). 2025;26(6):284–96. 10.2459/jcm.0000000000001733. PMC12404634 40472172

[29] Tang J, Ye L, Yan Q, Zhang X, Wang L. Effects of sodium-glucose cotransporter 2 inhibitors on water and sodium metabolism. Front Pharmacol. 2022;13:800490. 10.3389/fphar.2022.800490. PMC8905496 35281930

[30] Piperis C, Marathonitis A, Anastasiou A et al. Multifaceted Impact of SGLT2 Inhibitors in Heart Failure Patients: Exploring Diverse Mechanisms of Action. Biomedicines. 2024;12(10):2314. Published 2024 Oct 11. 10.3390/biomedicines12102314 PMC11504660 39457625

[31] Zelniker TA, Braunwald E. Mechanisms of Cardiorenal Effects of Sodium-Glucose Cotransporter 2 Inhibitors: JACC State-of-the-Art Review. J Am Coll Cardiol. 2020;75(4):422–34. 10.1016/j.jacc.2019.11.031. 32000955

[32] Duan S, Ma Y, Lu F, et al. High sodium intake and fluid overhydration predict cardiac structural and functional impairments in chronic kidney disease. Front Nutr. 2024;11:1388591. 10.3389/fnut.2024.1388591. Published 2024 May 27. PMC11164051 38860161

[33] Cardoso R, Graffunder FP, Ternes CMP, et al. SGLT2 inhibitors decrease cardiovascular death and heart failure hospitalizations in patients with heart failure: A systematic review and meta-analysis. EClinicalMedicine. 2021;36:100933. 10.1016/j.eclinm.2021.100933. Published 2021 Jun 5. PMC8257984 34308311

[34] Packer M, Zannad F, Butler J, et al. Influence of endpoint definitions on the effect of empagliflozin on major renal outcomes in the EMPEROR-Preserved trial. Eur J Heart Fail. 2021;23(10):1798–9. 10.1002/ejhf.2334. PMC9291539 34459076

[35] Yau K, Dharia A, Alrowiyti I, Cherney DZI. Prescribing SGLT2 Inhibitors in Patients With CKD: Expanding Indications and Practical Considerations. Kidney Int Rep. 2022;7(7):1463–76. 10.1016/j.ekir.2022.04.094. Published 2022 May 5. PMC9263228 35812300

[36] Palmer BF, Clegg DJ. Euglycemic Ketoacidosis as a Complication of SGLT2 Inhibitor Therapy. Clin J Am Soc Nephrol. 2021;16(8):1284–91. 10.2215/CJN.17621120. PMC8455044 33563658

[37] Lin DS, Lee JK, Chen WJ. Clinical Adverse Events Associated with Sodium-Glucose Cotransporter 2 Inhibitors: A Meta-Analysis Involving 10 Randomized Clinical Trials and 71 553 Individuals. J Clin Endocrinol Metab. 2021;106(7):2133–45. 10.1210/clinem/dgab274. 33895840

